# Insights into Chinese Canadian culture: enablers and barriers for fruit and vegetable intake

**DOI:** 10.3389/fpubh.2024.1349558

**Published:** 2024-04-23

**Authors:** Simran Gill, Debbie Lam, Natalie Choy, Anne Swann, Eric Liow, Tricia S. Tang

**Affiliations:** ^1^Centre for Chronic Disease Prevention and Management, Faculty of Medicine, University of British Columbia Okanagan, Kelowna, BC, Canada; ^2^Experimental Medicine, Faculty of Medicine, University of British Columbia, Vancouver, BC, Canada; ^3^Vancouver Coastal Health, Vancouver, BC, Canada; ^4^Department of Medicine, Faculty of Medicine, University of British Columbia, Vancouver, BC, Canada

**Keywords:** fruits, vegetables, Chinese-Canadian community, cultural differences, dietary education, nutrition

## Abstract

**Background:**

Fruits and vegetables (F&V) play a vital role in promoting health and preventing diseases. Numerous studies have demonstrated the association between F&V consumption and reduced risks of cardiovascular disease, cancer, and mortality. Despite the high priority of public health in promoting F&V intake, Chinese immigrants in Canada often fall below national guidelines in their consumption. Understanding the factors influencing F&V intake in this community is crucial for developing effective interventions.

**Methods:**

This study used an applied ethnographic research approach to gain insight into the enablers and barriers that influence F&V intake among Chinese-Canadian adults in Richmond, BC. Semi-structured interviews and ‘photovoice’ group sessions were conducted to gather qualitative data from community participants and health care providers (HCPs).

**Results:**

The research identified four key themes: (1) Cultural differences around how vegetables are perceived, consumed and prepared; (2) Motivators and strategies for increasing vegetable and fruit intake; (3) Lack of culturally relevant dietary education and resources; and (4) Importance of value in vegetable/fruit-related decisions. Participants showed a strong preference for the traditional Eastern diet, with cost of food and lack of knowledge about Western vegetables acting as barriers to dietary diversity. The study also highlighted the need for culturally tailored educational resources to effectively promote F&V consumption.

**Conclusion:**

By adopting a multi-modal approach, incorporating both interviews and ‘photovoice’ sessions, this research provided comprehensive insights into the participants’ perspectives and experiences related to F&V intake. Understanding these factors can guide the development of culturally appropriate interventions to increase F&V consumption among Chinese-Canadian adults in Richmond, BC, and potentially improve their overall health and well-being. Future studies should consider the heterogeneity within the Chinese immigrant population and target a more balanced representation of age groups to further enhance our understanding of F&V intake patterns in this community.

## Introduction

Fruits and vegetables (F&V) are a fundamental component of any diet and important in health promotion and disease prevention. Several studies, including systematic reviews and meta-analyses, have found F&V intake to be associated with a lower risk of cardiovascular disease (CVD), cancer, all-cause mortality (1–6), and impact psychological wellbeing (7, 8). Consistent with these findings, the Canadian Community Health Survey 2.2 found that consuming five or more daily servings of F&V was associated with reduced risk of overall chronic conditions (9).

In 2013, BC’s Guiding Framework for Public Health set a target to increase the percentage of the population 12 years and older who consume F&V at least 5 times per day from 44% in 2009–2010 to 55% in 2023 (10). Despite this public health priority to promote F&V consumption, the Canadian Community Health Survey reports in 2021, the number of people in BC who consume F&V 5 or more times per day decreased to 21% (11). Furthermore, a scoping review of 51 papers found that F&V intake, and fiber among Chinese immigrants in Canada and the United States fall below the national guidelines (12).

Among the numerous socio-economic and individual factors that influence dietary consumption, culture may also play a role. In fact, research has found acculturation from Eastern to Western lifestyles to have a negative impact on diet and health (13). For example, a scoping review (12) documented higher caloric intake, increased consumption of meat and alternatives, and elevated carbohydrate intake associated with acculturation (12). This trend may be partly explained by increase in portion sizes, more frequent dining out and higher consumption of convenience foods, as these behaviors were positively correlated to length of stay of Chinese immigrants in Canada (14). To date, the impact of acculturation on fruit and vegetable intake in this community has not been well-studied.

Richmond is home to one of the largest Chinese communities in Canada, accounting for 54% of the city’s total population (15), and therefore, an ideal setting to explore attitudes toward F&V intake in this ethnic group. Data from the 2013–2014 My Health My Community (MHMC) survey in Richmond found that 15% of Chinese respondents reported having 5+ servings of F&V per day, compared to 31% of Caucasian and 19% of South Asian respondents (16). Considering these data, the objectives of this study is to utilize a multi-modal qualitative research design to gain insights into enablers and barriers that influence F&V intake in the Chinese-Canadian community in Richmond, BC.

## Methods

This study was approved by the University of British Columbia’s behavioral research ethics board. To address our research questions and objectives, we applied the ethnographic research approach described by Hammerseley and Atkinson (17) which investigates individual’s experiences and behaviors in everyday context (i.e., photovoice) rather than under tightly structured conditions imposed by the researcher. Moreover, hypotheses generated are based on interpreting the motivation and outcomes of individuals’ behavior and how it is understood in their local environment.

### Sampling and recruitment

#### Community participants

To recruit community participants, we (1) posted bi-lingual flyers in community gathering spaces frequented by Chinese Canadians, (2) set up information tables at these same community sites to promote the study, (3) engaged ‘gatekeepers’ from community agencies to increase awareness, and (4) used word of mouth. To be eligible, individuals had to (1) be 18 years or older, and (2) self-identify as first-generation ethnic Chinese. Exclusion criteria were having a medical condition that limited F&V intake or having lived in Canada for less than 9 months. Interested individuals contacted the research team, learned more about the study, and completed informed consent procedures if they wanted to enroll. Upon completion, participants received a $5 gift card to a grocery store.

#### Health care providers (HCPs)

To recruit HCPs, we posted flyers at family physician or nurse practitioner offices, and engaged HCPs affiliated with Vancouver Coastal Health programs. HCPs were eligible if they (1) practiced in the Lower Mainland and (2) self-identified as having experience caring for Chinese-Canadian patients. Interested HCPs contacted the research team and were provided study information. Those interested completed informed consent procedures. Upon completion, HCPs received a $50 gift card.

#### Data collection

All community participants completed a survey assessing socio-demographic background (10 items: gender, age, languages spoken at home, city dwelling, employment status, education, marital status, country of birth, year of emigration, and years living in Canada) and answered questions regarding their fruit and vegetable intake and satisfaction with their food intake patterns.

A total of 48 community participants participated in either a semi-structured interview or two ‘photovoice’ sessions. Fourteen semi-structured interviews, approximately 60-min in length, were conducted. Interviewers were conducted in either English, Cantonese, or Mandarin. Additionally, 8 ‘photovoice’ sessions were held (2 sessions per group). Each group had 6–8 participants and the sessions were approximately 90 minutes in duration.

Thirteen health care providers participated in semi-structured interviews which were approximately 30 minutes in duration.

All interviews and ‘photovoice’ group sessions were recorded, then translated and transcribed directly into English for analysis. Each participant was provided with a unique study identifier. No participant identifiers were linked to any questionnaires or study transcripts. All recording files were encrypted and stored on Microsoft (MS) OneDrive, a secure file storage platform endorsed by the University of British Columbia (UBC).

#### Community participants

Following survey completion, community participants were given the choice to participate in semi-structured interviews or ‘photovoice’ group sessions. Both options were facilitated using an interview guide based on the Socio-Ecological Model (SEM) (18) and the Social Cognitive Theory (SCT) (19). Semi-structured interviews were conducted in either English, Cantonese, or Mandarin by the same researcher (NC)

‘Photovoice’ participants attended 2 meetings. The first meeting included a guided discussion on F&V intake, followed by an explanation of ‘photovoice’. Using guided questions, participants were asked to express their lived experiences around F&V intake through photography. They were invited to select 2 photographs to share with the group. They also completed 2 worksheets to convey additional thoughts and perspectives. At the second meeting, each participant shared their selected photographs and worksheets. A group discussion, facilitated by the researcher(s), followed each photograph presentation.

‘Photovoice’ group sessions were conducted in Cantonese or Mandarin by the same researcher (NC), while the English group sessions were conducted by 2 other researchers (AS and AB). To allow for the participation of English-speaking researchers, an interpreter was used to provide real-time interpretation so that other researchers could also understand and participate in the discussions.

#### Health care providers

HCPs completed semi-structured interviews in English by the same researcher (AS). Based on the SEM and the SCT, the HCP interview guide also sought to assess HCPs’ needs in supporting their patients to increase F&V intake.

#### Data analysis

All interviews and ‘photovoice’ group audio recordings were meticulously translated and transcribed into English to prepare for subsequent data analysis. A thematic analysis grounded in Braun and Clarke’s framework (20) was conducted. The team members familiarized themselves with the data through reading and re-reading of transcripts and reflective note taking. They then individually generated codes abductively until a unified codebook was agreed upon. Two coders (DL, SG) independently coded all transcripts using Nvivo R1 software. Any discrepancies were discussed until a consensus could be reached. Following coding, the team (DL, SG, TT) met to amalgamate themes and subthemes.

## Results

### Participant demographics: community participants

The number of participants taking part in interviews was 16; photovoice groups was 32. Participants were further divided into age cohorts: 14 were in the age 25–40 cohort, 18 were in the 40–60 cohort, 14 were in the 60 to 80 cohort and 2 were in the 80+ cohort. Participants were predominantly female, with 39 females and 9 males. Length of residency in Canada ranged from 1.3 years to 62 years. Countries of birth included Hong Kong (23), China (20), Taiwan (3), Singapore (1) and Vietnam (1).

### Participant demographics: health care providers

Thirteen health care providers took part in the interviews. There were 9 females and 4 males. Professions included physician (12) and dietitian (1). All were practicing in the Metro Vancouver area with 10 in Vancouver and 3 in Burnaby. Predominantly, they were Chinese in ethnicity (11 out of 13) and as a criterion to take part in the interviews, they all had depth of experience working with Chinese Canadians.

Through qualitative analysis, four overarching themes emerged: (1) Cultural differences around how vegetables are perceived, consumed and prepared; (2) Motivators and strategies for increasing vegetable and fruit intake; (3) Lack of culturally relevant dietary education and resources; and (4) Importance of value in vegetable/fruit-related decisions.

## Theme 1: cultural differences around how vegetables are perceived and consumed and prepared

### Subtheme A: Eastern versus Western dietary frameworks

There are clear distinctions in how food and eating are conceptualized between Eastern and Western culture. Not only do these differences pertain to traditional dishes prepared, but also, type of ingredients used, dietary habits practiced, as well as cooking techniques applied. For example, participants indicated that consuming raw (vs. cooked) vegetables is not a common practice in Chinese culture. In fact, the idea of having a “green salad” is not aligned with how many participants prepare and consume vegetables.

*“I know that you tried to encourage us to try raw vegetables in salad, for example kale. I’ve heard incidents when someone ate kale with worms and they went up to the person’s brain, which is very scary. I think we have to wash vegetables really thoroughly, which is why I am very scared of having raw vegetables.” –* Community member #8

Some participants adhere closely to an Eastern diet. Not surprisingly, their cupboards and refrigerators are stocked only with typical staples and ingredients necessary to make Chinese dishes. Given that cooking Eastern style is second nature for many participants, transitioning to a Western diet would involve substantial change and effort.

*“I rarely cook Western meals, mainly this is because I’m still not used to it. I feel that the ingredients in my home are not complete for making Western meals. So mostly I make fried rice – fried rice with an egg, put some beans [peas?], meat – all inside. As well, dumplings. Dumplings – fry it or boil, it doesn’t matter [both are ok]. And currently I’m used to, waking up in the morning, in the morning making rice and stir frying a dish. There’ll be vegetables and meat. So I’ll stir-fry a dish.” –* Community Member #2

As noted by participants and health care providers, the extent to which participants adopt an Eastern versus Western diet appears to be associated with the number of years living in Canada, as well as the generation (first generation, second generation) they identify with. Immigrants who had children born in Canada are more likely to become more acculturated with regards to dietary and cooking patterns.

*“Yes, my kids were born and raised here. And then we slowly … Our cooking style has also changed a bit. So now when cooking, it’s not exactly Chinese but not exactly Western, or say both Chinese and Western styles. If we cook Western food, they like it a lot. If we cook Chinese, then they uhh-uhhh [so-so, not great] But if we go out to eat at a Chinese restaurant, they think it’s tasty. Because what we cook is not as tasty as what the restaurant cooks.” –* Community Member #13

### Subtheme B: societal norms and living environments conducive for cooking at home

Although “eating out” is a social activity that most people are familiar with regardless of culture, the implications and circumstances around this practice are different when living in Asia versus Metro Vancouver. In Asia, the cost of “eating out” is similar to that of preparing food at home. Moreover, back in Asia “eating out” was more convenient given a busier day-to-day schedule. On the contrary, in Metro Vancouver, participants have the free time to prepare food at home and do not often eat out as it can be quite expensive.

*“I think we eat out more often in Hong Kong, because the price difference between eating out and cooking is about the same.” –* Community Member #6

Given the higher cost associated with eating out in Metro Vancouver, participants noted that they will opt to order fewer vegetables and more meat dishes to get more value for their money.

*“If, most of the times when we eat at a restaurant, we would usually order more meat. Because I think the restaurants here the meat and vegetable dishes have the same price! Vegetables are sold for $20 a plate and meat is also sold for $20 a plate. So how would you buy? Then you would order the meat.” –* Community Member #18

Health care providers noted that compared to the cramped living spaces in Asia, individuals have more spacious homes in Metro Vancouver, which may make cooking at home easier.

*“Another thing is that I guess it is cultural too but it depends on, maybe this one has to do with length of time in Canada, but the people who have recently moved, you know, they are still used to the fact that they used to live in 200 or 300 square foot family homes or apartments and many people in those situations, eating out is much more convenient than cooking in a very tiny kitchen and when you have to do clean up and everything.” –* Health Care Provider #1

### Subtheme C: lack of familiarity with specific vegetables and cooking techniques

Participants are hesitant to purchase Western vegetables because they do not how to prepare them well. Cooking western vegetables using eastern cooking techniques may not produce an appealing taste. To experiment with vegetables not commonly used, participants would need to learn different cooking approaches. However, learning new skills can be time consuming. It is a lot easier for the participants to purchase vegetables they are familiar with and cook them in a way that they know they will enjoy.

*“There are a lot that I haven’t cooked myself – for example squash, things like that. I haven’t cooked them before and don’t know how to season it. If I use my own methods of cooking vegetables, it might not be tasty.” –* Community Member #3

## Theme 2: motivators and strategies for increasing vegetable and fruit intake

### Subtheme A: motivators

Whether participants were willing to incorporate a specific vegetable or fruit into their diet hinged on two factors: (1) knowledge of the nutritional value and health benefits of the specific vegetable and/or fruit, and (2) an understanding of how the vegetable is prepared. These appeared to be important considerations when deciding whether to introduce the vegetable and/or fruit into one’s dietary repertoire. Moreover, participants were eager to share any information gleaned with their friends and other family members.

*“My suggestions are taking the photos of them, then list their benefits. Chinese all like to hear benefits if we eat [the vegetable or fruit] then they will buy them right away. Buy them instantly. For example, chia seeds – a lot of Chinese people don’t know it before, as soon as they heard it’s good, so they take tons of it home to China.” –* Community Member #1

For parents, a strong motivator for increasing vegetable intake pertains to the health and well-being of their children. Parents recognize that dietary patterns are formed early in childhood and that they serve as role models for their children. As such, parents want to be cognizant that the foods the family consumes are highly nutritious. Not only are they concerned about how fruits and vegetables are integrated into the daily diet, but also that their children are presented with a variety of choices. Not surprisingly, health care providers echoed the critical role of family environment in motivating children to adopt healthy dietary habits.

*“…by my mom, she basically cooks vegetables more…. This is because we were like that from when we were young until now” –* Community Member #4

### Subtheme B: strategies

Several strategies were attempted by participants with regards to increasing vegetable and fruit intake. In households where some family members were more carnivorous by nature, meat would be served alongside vegetables to encourage consumption of both foods. Although most participants utilize a traditional “family-style” model of dining, a few deliberately portioned out servings of vegetables to ensure that this food group was not overlooked by their children. However, this strategy is only successful when there is a mutual agreement between parents and children. If children are not amenable to this approach, parents often end up feeling frustrated. Finally, tactics to increase fruit consumption involve cutting up fruits ahead of time and serving it already prepared or packing fruits with the school lunch.

*“… [Everyday] I will prepare at least a plate of vegetables. And then I’ll give him a set amount. But…how do I say this? In the long term to do it like this, actually as a parent I will also have stress. I’ll eventually be faced with resistance, right? So then the amount that I can give, will not reach the amount that I feel I should give. I can only try.” -* Community Member #12

When examining factors that promote F&V intake, it would appear for participants that grew fruits and vegetables at home, they reported feeling confident about their own crop since it is both organic and easily accessible. For those who were novices to gardening, the idea of adopting such a hobby felt intimidating. However, when one participant shared personal stories around gardening (during a photovoice-based discussion), it clearly stimulated interest among others to grow their own fruit and vegetables.

*“Participant: I speaking about this photo – actually it was my backyard’s – last year’s summer harvest.*

*Participant: Harvest*

*Participants: WOW!*

*Participant: There are so many!*

*Participant: So if you say it matches your topic today. If you grow-grow things at home and the things [grown]- you like to eat, then for sure you would like it a lot and for sure will eat more. It’s like that.”*

## Theme 3: lack of culturally relevant dietary education and resources

According to several participants and health care providers, educational resources for healthy eating are not always culturally relevant. For instance, examples provided in nutrition pamphlets focus primarily on fruits and vegetables typically sold and consumed in North America, but not preferred by immigrants from Asia. Furthermore, merely translating documents from English to Chinese is not adequate. There is a need for dietary resources and learning opportunities to be culturally and linguistically tailored to Metro Vancouver’s Chinese community.

*“The challenge is that they may not be culturally adapted because they are pretty much direct translations of the English version.” –* Health Care Provider #3

*“If you still want to keep the plate, because it’s too much to do the bowl then throw some bok choy and all that other stuff that we normally eat in there.” –* Health Care Provider #7

Although the plate method is a great resource and dietary strategy for the Western diet, it does not accurately reflect how individuals from Asia eat and cook dishes. Whereas the Western diet conceptualizes meals into food groups, Eastern diet involves multiple food groups represented within a single dish. Additionally, meals are eaten “family style.”

*“If I cook a Chinese meal, then it’s all in the middle and you get it yourself. But if it’s a Western meal, then it’s all divided – a portion of steak and your portion of salad, I’ll put it on.” –* Community Member #9

Considering that Western style cooking is unfamiliar to individuals born and raised in Asia, participants indicated that it is important to make processes easy to follow. For example, when teaching the Chinese community how to cook healthily, recipes (especially for Western dishes) need to be simple and straightforward. Moreover, adapting recipes to accommodate more traditional Chinese ingredients would be another welcomed approach.

*“I will go on YouTube to look into. And then if I feel it is good, then next time I will buy it to try. If I find it’s too complicated or those that require adding a lot of Western seasonings in order to cook it then I might not.” –* Community Member #11

## Theme 4: importance of value in vegetable/fruit-related decisions

In the context of grocery shopping, fruit and vegetable purchases are substantially influenced by cost and freshness. However, if the cost of certain vegetables exceeds what is expected, this elevated price will likely factor into the decision-making process. In other words, the dietary choices are often times based more on economic value over nutritional value.

*“For example, I used to each spinach once a week, but now it’s getting expensive, so we don’t buy and eat.” –* Community Member #1

In particular, the price of fruits at the grocery stores are comparatively more expensive in Canada than participants’ home country. As such, participants are less likely to purchase fruits if they find that it is too expensive. The shopping patterns of many participants are based on what is on sale at the grocery store rather than their personal preferences.

*“Price, definitely. I really want to eat raspberry but the prices is really high. Sometimes it is very cheap, like $1-2. Then sometimes it is $4-5. I think. “Wow! Really, I can’t afford it!” –* Community Member #10

*“Yes, it will. It really will because nowadays fruits are expensive, so it really will. The other day I went to Kin’s Farm [Market] and the longans and cherries looked really delicious but they cost $10/lbs. so I didn’t buy them, so I’d rather eat an apple, but I don’t know if just eating apples are good.” –* Community Member #15

Participants often do their shopping at local Asian grocery stores, where they can purchase produce more commonly consumed by Chinese Canadians at a lower price. When asked if participants have frequented Farmer’s Markets, most of them have shopped in this setting. However due to the high prices, they tend to only go during the summer when fruits are offered at lower prices. While participants acknowledge the high quality of produce at Farmer’s markets, they still prefer shopping at cheaper and more convenient grocery stores.

*“Yes, I’ve bought before. Especially the beans, it taste especially good. Sometimes other vegetables … more expensive but if I just buy a little then it won’t seem that expensive. we’ll buy from there on occasion not every day.” –* Community Member #7

Cost and value also factor into eating outside the home. For example, in many Chinese restaurants, because the vegetable dishes and meat/fish dishes generally cost the same, participants are more likely to order the latter for better economic value. Moreover, participants can prepare vegetable dishes at home at a much lower cost. In Western restaurants, rather than ordering a separate vegetable dish, participants elect to get their recommended vegetable serving from the small portion that accompanies a main meat/fish entrée. The idea of paying extra for a serving of vegetables is not economical.

*“Boiled vegetables? Why would you order it from a Chinese restaurant when you can make it at home.” –* Community Member #16

## Discussion

This study explored enablers and barriers to healthy eating behaviors among Chinese adults living in the greater Richmond, BC area. Photovoice focus groups and semi-structured interviews identified four main themes: cultural differences around how vegetables are perceived, consumed, and prepared; motivators and strategies for increasing vegetable and fruit intake; lack of culturally relevant dietary education and resources; and importance of value in vegetable/fruit-related purchasing decisions.

Although participants have lived in Canada for an average of 20 years, there continues to be a strong preference for the traditional Eastern diet. This observation is consistent with a survey of 100 Chinese Canadian immigrants that reported 92% consume Chinese meals on a daily basis (21). Moreover, among a sample of 106 Chinese-Canadians, 60% chose to eat Chinese foods over Western foods and 44% perceived the former to be healthier than the latter (22). Traditional diets are not only perceived healthier, but also ingrained for so long that it is difficult to easily change (23, 24). Not only are Chinese-specific produce, cooking ingredients, and restaurants readily available and easily accessible in Richmond, but, grocery shopping at Eastern supermarkets is less expensive and more convenient. It would appear that, for this community, dietary patterns and purchases are driven largely by cost, value, and pragmatism.

Among the reasons participants are reluctant to explore the Western diet, lack of familiarity presents one obstacle. Participants tend to disregard Western fruits and vegetables that they do not know where to purchase or how to prepare. Similarly, Sanou and colleagues (25) also reported that individuals adhered to an Eastern diet due to the lack of knowledge with Canadian foods and its nutritional value. For example, frozen fruits and vegetables are not customary in Eastern diets and reported to be viewed as unhealthy and processed (26). However, frozen fruits and vegetables are commonly used in Western countries and serve similar nutritional value as the fresh versions (27, 28). Indeed, by restricting oneself to foods that are known to be safe and familiar, immigrants may forego opportunities that could expand their palette in a positive direction.

The lack of culturally relevant tools for nutrition education also discourages participants from making fully informed dietary decisions (29). Furthermore, attempts to create culture-specific resources cannot only be cosmetic in nature. In other words, translating existing educational materials from English to Chinese is not sufficient. To be effective, the fruits and vegetables presented in educational pamphlets, as well as its nutritional profiles, need to match what is actually consumed by the target audience (30). Equally important, teaching the community food preparation techniques for unfamiliar foods will also promote greater culinary experimentation (31).

Our findings suggest that strategies to motivate healthy eating patterns need to be family-based. Chinese families report that the desire to respect their families’ eating habits surpasses over individual preferences (32). In fact, according to a systematic review of communication between health professionals’ and Chinese immigrants, the collective well-being of the family is a key factor in decision-making (33). Not surprisingly, our interviews with health professionals also underscored the critical role of family in adopting healthy dietary habits. Clearly, efforts to improve dietary decisions and patterns in Richmond’s Chinese community needs to involve the family unit.

We adopted a comprehensive approach to explore fruit and vegetable intake patterns. Not only did we conduct semi-structured interviews, but this method was complemented by photovoice focus groups. Unlike semi-structured interviews, photovoice allows participants to “walk into the lives” of other community members and exchange experiences around grocery shopping, gardening, and eating outside the home. For example, participants who were initially unfamiliar with growing their own fruits and vegetables subsequently expressed enthusiasm to embark on such a project. Clearly, this type of inspiration is less likely to be elicited in a traditional semi-structured interview. However, interviews were also essential to explore the attitudes, beliefs, and perceptions around fruits and vegetables of each individual. Indeed, utilizing both methods allowed for more rich and robust data collection.

This study is not without limitations. Richmond’s Chinese is comprised of individuals originating from Hong Kong, China, Taiwan, Singapore, and Vietnam. Thus, not only are dietary patterns influenced by factors such as the number of years living in Canada, but also by regional and geographical differences. For example, there are differences with regard to spices and ingredients by region (34, 35). Given this heterogeneity, our results may not adequately reflect any single cultural group represented. Additionally, considering that 71% of our sample were 40 years and older, the dietary perspective, habits, and patterns of young adults are not well reflected. Although our interview guide was designed to query for both fruit and vegetable intake, discussions often veered toward the latter. Future studies should intentionally steer interviews to encourage more balanced content.

## Conclusion

This study provides valuable insights into the enablers and barriers influencing fruit and vegetable intake among Chinese adults in the Richmond, BC area. Through a multi-modal qualitative research approach consisting of semi-structured interviews and photovoice focus groups we note a preference for traditional Eastern diet among Chinese immigrants. Multiple factors contribute to the dietary acculturation process including the availability of and access to fresh fruits and vegetables, education and familiarity of different food types and preparation styles, food costs/values, environmental conditions, etc. By understanding the factors associated to dietary decisions and patterns, we can develop more effective strategies and interventions to increase fruit and vegetable intake.

## Data availability statement

The original contributions presented in the study are included in the article/supplementary material, further inquiries can be directed to the corresponding author.

## Ethics statement

The studies involving humans were approved by The University of British Columbia Behavioural Research Ethics Board. The studies were conducted in accordance with the local legislation and institutional requirements. Written informed consent for participation in this study was provided by the study participants.

## Author contributions

SG: Formal analysis, Methodology, Writing – original draft. DL: Formal analysis, Methodology, Writing – original draft. NC: Conceptualization, Investigation, Writing – review & editing. AS: Conceptualization, Investigation, Writing – review & editing. EL: Writing – review & editing, Project administration, Supervision. TT: Supervision, Writing – review & editing, Conceptualization.
